# Type Three Secretion System in *Pseudomonas savastanoi* Pathovars: Does Timing Matter?

**DOI:** 10.3390/genes2040957

**Published:** 2011-11-25

**Authors:** Stefania Tegli, Andrea Gori, Matteo Cerboneschi, Maria Grazia Cipriani, Angelo Sisto

**Affiliations:** 1 Laboratorio di Patologia Vegetale Molecolare, Dipartimento di Biotecnologie Agrarie, Universitá degli Studi di Firenze, Via della Lastruccia 10, 50019 Sesto Fiorentino, Firenze, Italy; E-Mails: andr.gori@gmail.com (A.G.); matteo.cerboneschi@unifi.it (M.C.); 2 Plant Protection Institute, Section of Bari, National Research Council (CNR), Via Amendola 122/D, 70126 Bari, Italy; E-Mail: mg.cipriani@ipp.ba.cnr.it; 3 Institute of Sciences of Food Production (ISPA), National Research Council (CNR), Via Amendola 122/O, 70126 Bari, Italy; E-Mail: angelo.sisto@ispa.cnr.it

**Keywords:** Type Three Secretion System, *hrp*, *Pseudomonas syringae*, *Pseudomonas savastanoi*, evolution, recombination, gene expression, regulation

## Abstract

*Pseudomonas savastanoi* pv. *savastanoi* is the causal agent of Olive knot disease, relying on the Type Three Secretion System (TTSS) for its pathogenicity. In this regard, nothing was known about the two other pathovars belonging to this species, pv. *nerii* and pv. *fraxini*, characterized by a different host range. Here we report on the organization of the entire TTSS cluster on the three pathovars, and a phylogenetic analysis including the TTSS of those bacteria belonging to the *P. syringae* complex sequenced so far, highlighting the evolution of each operon (*hrpC*, *hrpJ*, *hrpRS*, *hrpU* and *hrpZ*). Moreover, by Real-Time PCR we analyzed the *in vitro* expression of four main TTSS genes, revealing different activation patterns in the three pathovars, hypothetically related to their diverse virulence behaviors.

## 1. Introduction

The Type III Secretion System (TTSS) is a multi-molecular device which is essential for the pathogenicity of Gram-negative bacteria infecting plants, animals and humans, which use this system for the one-step delivery of effector proteins from the bacterial cytoplasm into that of the eukaryotic host cells.

It has been widely demonstrated that in plant pathogenic bacteria, both the triggering of the hypersensitive response (HR) in non-host or host resistant cultivars, and the pathogenicity on the susceptible cultivars of the hosts, strictly depends on the correct functionality of their TTSS. For this reason, the genes coding for the TTSS of phytopathogenic bacteria are indicated by the acronym *hrp* (*hypersensitive response* and *pathogenicity*). The additional acronym *hrc* is used for those *hrp* genes highly conserved in the TTSSs of bacterial pathogens of plants and animals [1].

According to the most recent model drawn for the bacteria belonging to the *Pseudomonas syringae* complex, at the beginning of the bacterium-plant interaction, specific effectors secreted via TTSS are involved in the suppression of the plant basal defences, which are elicited by the so called Pathogen or Microbe-Associated Molecular Patterns (PAMPs or MAMPs) and named PAMP-Triggered Immunity (PTI) [2-5]. On the other hand, when injected into the cytoplasm of a resistant cultivar of the host, TTSS effectors can be individually recognized by intracellular Nucleotide-Binding site, Leucine-Rich Repeat (NB-LRR) Resistance proteins (R), activating a second line of defence known as Effector-Triggered Immunity (ETI) [2-5] characterized by the development of HR. Various models have been recently elaborated to explain the molecular mechanisms at the basis of this recognition. As far as the susceptible hosts are concerned, the disease occurs through the activities of those TTSS effectors which are not recognized by host R proteins or able to suppress ETI [2-6]. In several phytopathogenic bacteria belonging to the *P. syringae* complex, the expression of *hrp*/*hrc* genes has been demonstrated to be triggered and regulated by both environmental and host factors [7-12]. *In vitro* expression of these genes is low or partially repressed when bacteria are grown in nutrient-rich media, whereas it rapidly increases when bacteria are grown in minimal medium, with or without plant cell exudates added, or are infiltrated into host tissues [7-9,11-16].

The regulation of TTSS in bacteria of the *P. syringae* complex has been described and dynamically modeled. Within this regulatory network HrpRS, HrpA, HrpV play an essential role and HrpL is one of the most important information processing points [11,17,18]. Moreover, critical linkages were found between TTSS transcriptional regulators and several global signal transduction systems [11,14,19-22].

*P. savastanoi* pv. *savastanoi* (*Psv*) was demonstrated to fully rely on the functionality of its TTSS to successfully induce knot formation in Olive trees [23,24]. So far, no information is available on this system for the other two pathovars belonging to this species, *nerii* (*Psn*) and *fraxini* (*Psf*), which attack woody plants as well, Oleander and Ash respectively. Here, for the first time, we report the whole sequence and organization of the TTSS clusters of *Psn* and *Psf*, together with that of a well-known *Psv* strain. These data were used to determine the phylogenetic position of these pathogens into the *P. syringae* complex, using the *hrp* clusters fully sequenced until now in these bacteria. Moreover, for the first time to our knowledge, through Real-Time PCR we analyzed the kinetics of the *in vitro* expression of TTSS in *Psv*, *Psn* and *Psf*, to investigate if the timing and/or the wiring of TTSS regulation could have any potential concerning the evolution of virulence across these *P. savastanoi* pathovars.

## 2. Results and Discussion

### 2.1. Organization of TTSS Cluster in P. savastanoi Pathovars

**Figure 1:**
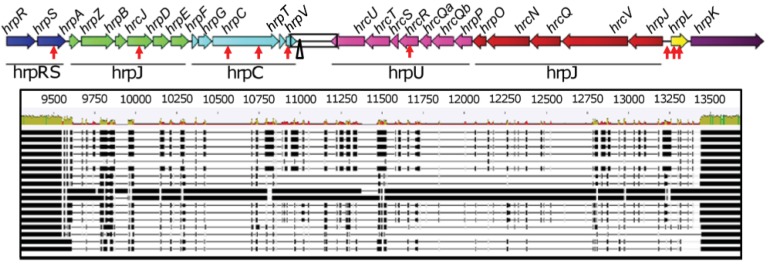
Organization of Type III Secretion System (TTSS) cluster of *Psv*5, *Psn*23 and *Psf*134. Red arrows represent the single nucleotide polymorphisms (SNPs) found among pathovar TTSS sequences. Black triangle indicates the localization of a partial insertion sequence, remnant of an IS66 element. Names of operons are not in italics to differentiate from gene names. For the part of the sequence highlighted by the black square, corresponding to the hypervariable region between *hrp*C and *hrp*U operons, the alignment among the sequences derived from the seventeen *Pseudomonas* species examined in this study is schematically reported. These species are ordered from top to bottom as: (1) *P. savastanoi* pv. *savastanoi* ITM317 (*Psv*5); (2) *P. savastanoi* pv. *nerii* ESC23 (*Psn*23); (3) *P. savastanoi* pv. *fraxinii* NCPPB1006 (*Psf*134); (4) *P. syringae* pv. *aesculi* str. 2250; (5) *P. syringae* pv. *phaseolicola* 1448A; (6) *P. syringae* pv. *tabaci* ATCC11528; (7) *P. syringae* pv. *syringae* str. 61; (8) *P. syringae* Cit 7; (9) *P. syringae* pv. *syringae* B728a; (10) *P. syringae* pv. *aceris* M302273PT; (11) *P. syringae* pv. *pisi* 1704B; (12) *P. syringae* pv. *aptata* DSM50252; (13) *P. syringae* pv. *japonica* M301072PT; (14) *P. syringae* pv. *tomato* DC3000; (15) *P. syringae* pv. *tagetis* LMG5090; (16) *P. viridiflava* PNA3.3a; (17) *P. viridiflava* LP23.

The organization of the TTSS clusters of *Psv5* (FR717896), *Psn23* (FR717897) and *Psf134* (FR717898) was discovered to be identical among these pathovars as shown in Figure 1.

The TTSS cluster is 23,835 bp long, and it is composed of twenty-seven genes, most of which arranged in five operons organized in two main blocks having convergent genes transcription (*hrpRS*, *hrpZ* and *hrpC* on one hand, and *hrpU* and *hrpJ* on the other, conventionally reported on the right and left side of the cluster in Figure 1). These two blocks are separated by an hypervariable region, with a very low level of conservation between closely taxonomically related bacteria and containing a remnant of an insertion sequence of the IS66 family [25] (Figure 1). The two genes *hrpL* and *hrpK*, coding for a sigma-54 factor [26] and for a protein involved in translocation [27] respectively, are not included in any operon. This kind of organization is known to be shared among other bacterial phytopathogens, like *P. syringae*, having the so called “Group I Hrp TTSS” [28,29]. The GC content of the entire TTSS cluster of *Psv5*, *Psn23* and *Psf134* is 58.8%. The same value was calculated for TTSS cluster of *Psv* strain NCPPB3335, whose genome was reported to have a GC% content appreciably lower (57.1%) [30]. In all the three *P. savastanoi* pathovars examined here it was demonstrated that the TTSS cluster is chromosomally located and that each gene is present in a single copy, as assessed by Southern blot and Real-Time PCR, respectively (data not shown).

As far as TTSS sequences of *Psv5*, *Psn23* and *Psf134* are concerned, just nine Single Nucleotide Polymorphisms (SNPs) were found to be differentially present in the clusters of these bacteria, eight of which located into an open reading frame (ORF) (Figure 1). In Table S1 and S2 the characteristics of the putative proteins coded by *hrp/hrc* genes are reported [31-36]. This bioinformatic analysis encourages hypothesizing that these SNPs do not affect the presence of conserved domains on predicted proteins and their subcellular localization, even when non-synonymous mutations were considered. In the same tables, the homologies between Hrp/Hrc predicted proteins of *P. savastanoi* and of *P. syringae* pv. *phaseolicola* 1448A are reported as well. These values range between 97% and 100%, to farther support the close relationship between these bacteria and their Hrp systems.

### 2.2. Phylogenetic Analysis of TTSS Cluster in P. savastanoi and Other Species of *P. syringae* Complex

Fourteen different *P. syringae* pathovars or closely related species, whose TTSS clusters have been completely sequenced, were selected (Table 1) [37-45] to be included in a global phylogenetic sequence analysis together with the three *P. savastanoi* pathovars previously mentioned. Comparisons were carried out analyzing both the entire TTSS cluster and each operon separately, in order to better understand the fundamental steps driving the evolution of TTSS in these bacteria.

**Figure 2:**
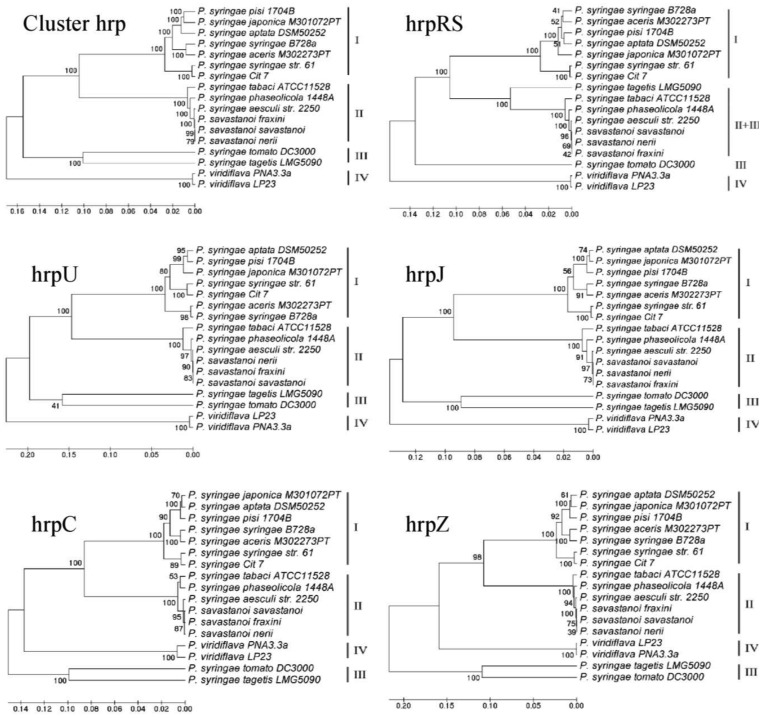
ML phylogenetic trees of TTSS cluster and of five single operons. The operon referred to each tree is indicated above each image. Groups are numbered from I to IV, and are related to the tree obtained analyzing the entire TTSS cluster. The trees are drawn to scale, with branch lengths measured in the number of substitutions per site. The congruence between trees and data (SH test) is reported in Table 2. “*P. savastanoi savastanoi*”, “*P. savastanoi fraxini*” and “*P. savastanoi nerii*” are strains ITM317, NCPPB1006 and ESC23, respectively. Names of operons are not in italics to differentiate from gene names.

In Figure 2 and in Table 2 the results obtained are shown and statistically evaluated, respectively. The analysis of the entire TTSS cluster and of each operon generated four major branches, with the three *P. savastanoi* pathovars always in the same minor branch, because of their very small sequence differences. The composition of three of the major branches, corresponding to Groups I, II and III, was coherent with data already obtained using *hrpS* and *hrpL* genes [46] or the single *hrpZ* operon [47]. For the first time the composition of these groups and their organization can be definitely confirmed both analyzing the entire TTSS cluster and using a representative range of species of the *P. syringae* complex. Here in Group I, only the bacteria belonging to genomospecies 1 were included [48,49]. Similarly, Group IV was exclusive of *P. viridiflava*, which was the only one in this analysis belonging to genomospecies 6. The three *P. savastanoi* pathovars examined here were always included in Group II, together with *P. syringae* pv. *aesculi*, another phytopathogenic bacterium attacking woody plants. Other bacteria belonging to genomospecies 2 were also clustered in Group II, such as *P. syringae* pv. *tabaci* and pv. *phaseolicola* [48,49]. As far as *P. syringae* pv. *tomato* and pv. *tagetis* are concerned, although belonging to genomospecies 3 and 7 respectively [48,49], here they clustered in Group III. The only exception was the phylogenetic tree derived from the analysis of operon *hrpRS*, where Group III was split and pv. *tagetis* was on a minor branch closely related to that of Group II. Moreover, a change occurred in the topology of the phylogenetic trees obtained from *hrpZ* and *hrpC* operons towards those referred to the entire TTSS cluster and to *hrpJ* and *hrpU* operons, with the inversion of Groups III and IV (Figure 2).

**Table 1:** Bacterial strains used for TTSS phylogenic analysis.

**Strain**	**Accession Number a**	**References**
*P. savastanoi* pv. *fraxinii* NCPPB1006 b	FR717898	This work
*P. savastanoi* pv. *nerii* ESC23 b	FR717897	This work
*P. savastanoi* pv. *savastanoi* ITM317 b	FR717896	Sisto *et al.*, 2004 [23]
*P. syringae* Cit 7	GL385012	Baltrus *et al.*, 2011 [37]
*P. syringae* pv. *aceris* M302273PT	GL385308	Baltrus *et al.*, 2011 [37]
*P. syringae* pv. *aesculi* str. 2250	NZ_ACXT01000080	Green *et al.*, 2010 [38]
*P. syringae* pv. *aptata* DSM50252	GL385257	Baltrus *et al.*, 2011 [37]
*P. syringae* pv. *japonica* M301072PT	GL384839	Baltrus *et al.*, 2011 [37]
*P. syringae* pv. *phaseolicola* 1448A	CP000058	Joardar *et al.*, 2005 [39]
*P. syringae* pv. *pisi* 1704B	GL384897	Baltrus *et al.*, 2011 [37]
*P. syringae* pv. *syringae* B728a	CP000075	Feil *et al.*, 2005 [40]
*P. syringae* pv. *syringae* str. 61	EF514224	Alfano *et al.*, 2000 [41]
*P. syringae* pv. *tabaci* ATCC11528	FJ946987	Studholme *et al.*, 2009 [42]
*P. syringae* pv. *tagetis* LMG5090	DQ246442	Song *et al.*, 2005 [43]
*P. syringae* pv. *tomato* DC3000	AE016853	Buell *et al.*, 2003 [44]
*P. viridiflava* LP23	AY597277	Araki *et al.*, 2006 [45]
*P. viridiflava* PNA3.3a	AY597278	Araki *et al.*, 2006 [45]

a Accession numbers are referred to their TTSS sequence or, in case of availability, to their complete genome;

b Codes used at LPVM for strains ITM317, NCPPB1006 and ESC23 are *Psv5*, *Psf134* and *Psn23*, respectively.

**Table 2:** SH test.

**Tree**							

		**hrpRS**	**hrpZ**	**hrpC**	**hrpU**	**hrpJ**	**hrpTOT** b
Data(seq)	hrpRS logL	−8024.7	−**8317.7**	**8258.7**	**−8263.5**	**−8300.4**	**−8259.1**
	ΔlogL	BEST	−**292.9**	**−234.0**	**−238.7**	**−275.7**	**−234.3**
	P value a	-	**0.000**	**0.000**	**0.000**	**0.000**	**0.000**
	hrpZ logL	**−20247.2**	−18951.0	−18968.5	−18980.4	−18977.6	−18983.7
	ΔlogL	**−1296.2**	BEST	−17.5	−29.5	−26.7	−32.7
	P value a	**0.000**	-	0.539	0.382	0.408	0.371
	hrpC logL	**−14691.8**	−14011.4	−13904.6	−13935.3	−13926.7	−13917.5
	ΔlogL	**−787.1**	−106.8	BEST	−30.6	−22.0	−12.8
	P value a	**0.000**	0.053	-	0.383	0.476	0.654
	hrpU logL	**−20969.1**	−**20475.6**	−20327.7	−20314.6	−20324.7	−20328.3
	ΔlogL	**−654.5**	**−161.0**	−13.1	BEST	−10.1	−13.7
	P value a	**0.000**	**0.005**	0.644	-	0.681	0.637
	hrpJ logL	**−26050.1**	**−25554.7**	−25361.1	−25387.2	−25354.8	−25363.5
	ΔlogL	**−695.3**	**−199.9**	−6.3	−32.4	BEST	−8.7
	P value a	**0.000**	**0.003**	0.824	0.408	-	0.755
	hrpTOT b logL	**−121132.6**	**−117951.1**	−117292.9	−117348.7	−117362.0	−117158.7
	ΔlogL	**−3973.9**	**−792.4**	−134.2	−190.0	−203.3	BEST
	P value a	**0.000**	**0.000**	0.202	0.107	0.094	-

a P-value identifies the probability that the data fits the tree. Bolded values indicate trees that fit the data significantly worse than the best tree given the data. Names of operons are not in italics to differentiate from gene names;

b hrpTOT refers to the tree or the data obtained from the analysis of the entire TTSS cluster.

More important considerations can be raised following a deeper statistical analysis carried out performing the Shimodaira-Hasegawa (SH) test [50], aimed to determine if the phylogenetic trees obtained by analysing the entire TTSS or any of its operons are as good as the best trees derived from each of the different data sets. In other terms, SH test ascertains if different data sets share the same evolutionary history. Therefore, the significance of the topological differences among *hrp* operons and the whole cluster was tested and evaluated to understand their contribution to the global phylogenetic variation of TTSS. The results obtained are reported in Table 2. The operon *hrpU* and *hrpJ* data were found to be congruent with all but the *hrpRS* and *hrpZ* trees. The operon *hrpZ* and *hrpC* data were found to be congruent with the tree generated from the whole TTSS cluster and with the trees of all the other operons but *hrpRS*'. In this regard, a remnant of an insertions sequence was found in the hypervariable region between *hrpC* and *hrpU* operons of the three *P. savastanoi* pathovars here examined, as previously reported in the TTSS clusters of other *P. syringae* pathovars [41,42,51]. Together these data seem to suggest that an important recombination event has occurred early in the evolution of TTSS cluster in the *P. syringae* complex, that it was more likely to happen via Horizontal Genetic Transfer (HGT) from an enteric bacterium pathogenic on animals or humans [52,53]. The direction of this hypothetic HGT event is also suggested by the different genomic localization of the TTSS clusters in pathogenic bacteria of animals and plants. Mainly these clusters are located on plasmidic pathogenicity islands (PAI) among the enteric bacteria pathogens for animals and humans, while they are often integrated into the bacterial chromosome in those pathogenic on plants [53,54]. Moreover, it is worth noticing that when the architecture of *P. savastanoi* TTSS was compared with that of enterobacterial phytopathogens, belonging to Group I Hrp TTSS cluster with *P. syringae*, genes of *hrpC* and *hrpZ* operons appeared to be transposed upstream of *hrpL* gene, and *hrpJ* and *hrpU* operons, though maintaining their orientation [54]. In accordance with all these data, the most widely accepted hypothesis is that a common ancestor of *P. syringae* and of enterobacterial plant pathogens likely acquired TTSS cluster by a single HGT prior to their speciation [53]. Then multiple interspecific HGT events would have occurred during their evolution, both among *P. syringae* and the enteric phytopathogens. The acquisition of information essential to allow the adaptation of these pathogens to new hosts were stably maintained in their genomes, thus explaining the incongruences found among the phylogenies of TTSS genes and operons [53].

Another important finding derived from the SH test refers to the operon *hrpRS*. Here for the first time we demonstrated the complete incongruence between operon *hrpRS* data and any of the trees obtained except its own. The *hrpS* and *hrpR* genes are two of the most important regulators of TTSS physiology. HrpS and HrpR proteins are supposed to act as a dimer to promote the expression of *hrpL*, coding for a sigma-54 factor [17]. Our data seem to fit the theory recently hypothesized by Jovanovich and colleagues [55] assessing that the operon *hrpRS* was crucial in the evolution and the expression of pathogenicity/virulence in these bacteria. According to their data, it seems that a co-evolution of *hrpR* and *hrpS* genes has occurred, probably following a gene duplication event during the *P. syringae* adaptive evolution to its different hosts. Besides TTSS regulatory elements, this evolution appears to have been positively driven also by several mutations involving bacterial effectors. This was very recently assessed by profiling the TTSS effectors of bacteria belonging to the *P. syringae* complex [56], with results highly consistent with those obtained here. Together these data suggest the hypothesis that during the patho-adaptive differentiation of the *P. syringae* complex, elements of the flexible genome follow an evolution strictly consistent with that of the core genome [57]. To support this hypothesis, a common evolutionary history has already been reported for two TTSS regulatory genes, among which *hrpS*, and the two housekeeping genes *gyrB and rpoD* [46]. If this was further confirmed, phylogenetic studies focused on TTSS would make an important contribution in the determination of the evolutionary relationships among bacteria of the *P. syringae* complex. In this regard, our data confirmed previous findings about *hrpZ* operon [24,47], that was proved to be the best candidate to unveil the evolutionary shaping of TTSS, together with *hrpC* operon. Moreover, the potential of *hrpRS* operon to display the patho-adaptive forces acting on this system was also revealed.

### 2.3. Time Course Expression Analysis of TTSS in P. savastanoi Pathovars

According to the recent findings concerning the importance of TTSS regulatory genes, such as *hrpS*, in the adaptive evolution of these bacteria to their hosts [55], for the first time we also investigated the time course expression of several TTSS genes of the three *P. savastanoi* pathovars here examined. This analysis was carried out by Real-Time PCR on cells grown *in vitro* on *hrp*-inducing minimal medium (MM) [7] and on nutrient-rich medium King's B medium (KB) [58]. To this purpose, *hrpS, hrpL* and *hrpV* genes were selected, the last two coding for a positive and a negative regulator of HrpS-promoted TTSS transcription, respectively [17]. Together with these genes, the structural gene *hrpA*, coding for the main protein subunit of the TTSS pilus, was also tested [17]. Using the primers reported in Table 3, amplification efficiencies ranged from 95% and 105%. The efficiency of the primer pairs for *hrpA*, *hrpL*, *hrpS* and *hrpV* genes was less than 5% different from that of the primer pair for the housekeeping gene *16S rDNA*, used for data normalization [59]. At an annealing temperature of 60 °C, efficiency values for *hrpA*, *hrpS*, *hrpL* and *16S rDNA* were 100.7%, 100.3%, 98.5% and 100.2%, respectively. At an annealing temperature of 62 °C, the values for *hrpV* and *16S rDNA* were 99.6% and 100.2%, respectively. The normalized relative expression of each gene was analyzed in MM *vs.* KB medium grown cells, at 3, 6, 18 and 24 h after inoculation of a starter culture in the corresponding fresh medium.

**Table 3:** Primer used in Real-Time PCR.

**Gene name a**	**Primer name**	**Primer sequence (5′ to 3′)**	**Amplicon size (bp)**
*hrpA*	hrpA RT for	GCAGGGTATCAACAGCGTCAAG	156
	hrpA RT rev	CCGTTCTCTTCGTTCGCAGT	
*hrpS*	hrpS RT for	AGCGGCACAAGGCGGAAC	156
	hrpS RT rev	TGGGCCGAAGCGATCACG	
*hrpL*	hrpL RT for	AGCCGCAGACCTGGTTGTG	159
	hrpL RT rev	ATTGCCTGTGCCCGTCTACC	
*hrpV*	hrpV RT for	CGTCCCGAGCAACTGAGAGAG	162
	hrpV RT rev	ATGTCGCCGTATGTCATCCAGG	
*16S rDNA*	16s RT for	GGAATCTGCCTGGTAGTGGGG	157
	16s RT rev	GGCTCACCAAGGCGACGAT	

a Gene targeted by the corresponding primer pair.

**Figure 3:**
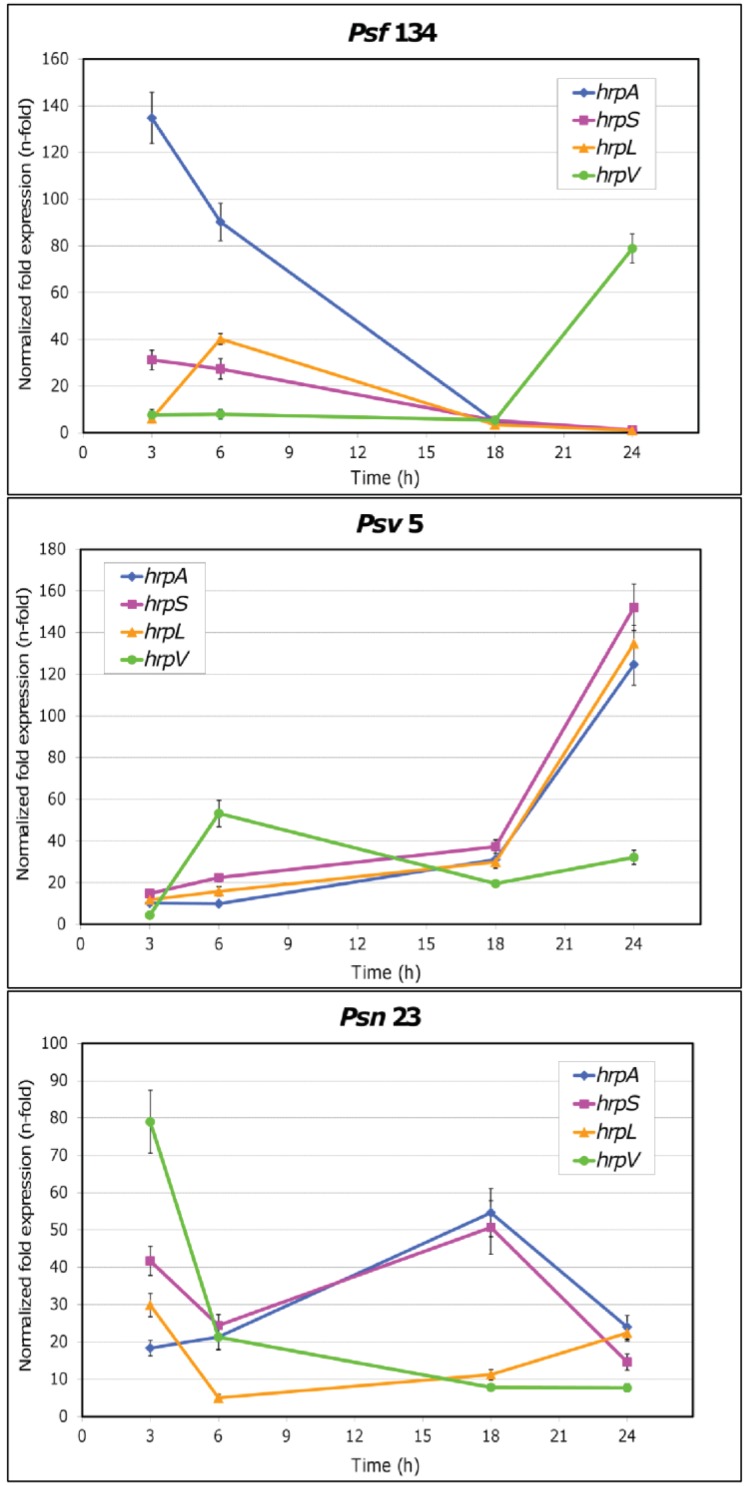
Time course of *hrpL*, *hrpA*, *hrpS* and *hrpV* expression in *P. savastanoi* pv. *fraxinii* (*Psf134*), pv. *savastanoi* (*Psv5*) and pv. *nerii (Psn23*). Relative expression in minimal medium (MM) is reported, setting expression in King's B medium (KB) as reference [59]. The pathovar referred to each graph is reported as graph title. The color used for each gene is indicated in the label. The vertical lines indicate standard deviation values, and where they are absent, the limits were within the symbol dimensions. The numerical values for each point analyzed are reported in Table S3.

The results obtained are reported in Figure 3 and in Table S3. For the first time it was demonstrated that TTSS genes are overexpressed in cultural conditions mimicking the plant apoplast in each of the three *P. savastanoi* pathovars here examined. Moreover pathovar-specific expression patterns were observed. In particular, the highest values for the relative expression of both *hrpA* and *hrpS* were reached by *Psf134* after three hours, while *hrpL* peaked at six hours (Figure 3). The expression of all genes dropped off after 18 h with the exception of *hrpV*, supposed to be an inhibitor of HrpS action [12,17], that increased up to a level of 80 fold after one day from the beginning of the simulated infection. Different expression patterns were shown by *Psv5* (Figure 3). Relative expression levels of *hrpA*, *hrpL* and *hrpS* remained low for the first 18 h of growth on MM, after which they started to rise and reached levels ranging from 124.7 to 152.1 fold at 24 h. As expected, *hrpV* followed an opposite trend, since its highest relative expression level was reached after 6 h of bacterial growth on MM (53.2 fold). As far as *Psn23* is concerned, the expression pattern of TTSS genes seemed to be more finely tuned (Figure 3). In particular, the highest relative expression levels for *hrpA* and *hrpS* were reached after 18 h, and then they started to decrease. The genes *hrpV* and *hrpL* peaked at 3 h of growth on MM. While *hrpV* signals progressively decreased until the end of the experiment, *hrpL* had a slight but continuous recovery up to 24 h (22.4 fold).

Several models have been proposed to describe the regulatory network for *hrp/hrc* gene expression in *P. syringae* and other phytopathogens, according to data collected in experiments mainly carried out *in vitro* using different *hrp*-inducing media and less frequently *in planta* [7-17,19-22,60]. These models are considerably variable among different bacterial phytopathogens even when closely related. However the importance of HrpL as a central point within this network and for the transcription of the other TTSS genes is constantly reported, as confirmed very recently by Boolean model simulating the activity of the *hrp* regulon in *P. syringae* [18]. Furthermore this model gave an important although theoretical contribution to clarify some conflicting data concerning the role of GacS/GacA system on the dynamical regulation of TTSS gene expression [12,61]. According to this model, the TTSS appears to be very tightly regulated, following the sequence of events rather than the timing as the most critical regulating factor. This would guarantee a more robust stability to any casual perturbation, such as evolutionary changes in the sequences of any TTSS components involved. In this frame GacS/GacA is here indicated as the only determinant of the expression of *hrp* regulon, although some important points still need to be elucidated. Among these there are the biotic and abiotic signals perceived by GacS sensor kinase, the production of the response regulator GacA, the mode of interaction between GacS and GacA, and the integration of this system with other regulatory elements known to act on *hrp* regulon. To this concern, a main role was demonstrated to be played by Lon protease, essential for switching on and off the *P. syringae* TTSS genes in inducing and repressive conditions, respectively [12,21].

Our results clearly suggest that the *in vitro* expression of TTSS is differently regulated in the three *P. savastanoi* pathovars here examined. A pathovar-specific regulation of *hrp*/*hrc* genes could be hypothesized to occur also *in planta*, matching the environmental conditions found by these *P. savastanoi* pathovars in their respective hosts, and possibly having some role in host-range determination. Intriguingly, in the first hours of growth on *hrp*-inducing medium the overexpression of the gene coding for the negative regulator HrpV was higher in those pathovars having the broadest host ranges, which are *Psn* and *Psv*. In contrast in *Psf*, which is able to attack just Ash, the early and apparently HrpV-unrestricted overexpression of *hrpA* could contribute to narrow its host range. Until now there is no clear evidence of any elicitor activity for HrpA. However this protein is the main component of TTSS pilus and its coding gene was demonstrated to be under strong diversifying selection into *P. syringae* species, presumably to avoid recognition by the host [47].

Nothing is known yet about regulatory systems acting upstream to TTSS, such as GacS/GacA and Lon protease, in none of the *P. savastanoi* pathovars here studied. Therefore it cannot be ruled out that their pathovar-specific expression patterns for the *hrp* genes examined could depend on their different abilities to perceive and translate environmental stimuli, such as those given by *hrp*-inducing medium. Actually differential modes for *hrpR* and *hrpS* expression were already found in *P. syringae* pv. *syringae* and pv. *tomato* [62-64], as well as for their regulation by GacS/GacA [61]. Furthermore, some of the SNPs we found in the TTSS sequences of these *P. savastanoi* pathovars mapped in the regulatory genes *hrpS*, *hrpL* and *hrpV*. It is worth noticing that just in these cases these SNPs gave rise to changes in the coiled-coil regions of the corresponding proteins (Table S1 and Table S2). Coiled-coil regions are known to be essential in several protein-protein interactions, particularly in the formation of multimeric complexes and in molecular recognition events [65,66].

Further experiments need to be carried out to prove if the pathovar-specific expression of *hrp/hrc* genes in *P. savastanoi* is dependent on a differential regulation of this system and if coiled-coil interactions are involved.

## 3. Experimental Section

### 3.1. Bacterial Strains and Growth Conditions

The *P. savastanoi* strains used in this study are *Psv*5, *Psn*23 and *Psf*134, belonging to the pathovars *savastanoi*, *nerii*, and *fraxini* respectively (Table 1). They were routinely grown at 26 °C, on KB [58] or on MM [7], in solid or in liquid cultures according to the experimental purposes.

Bacterial growth was monitored by determining the optical density at 600 nm (OD_600_) at different times during incubation, and bacterial concentration was estimated by serial dilutions and plate counts. For long-term storage, bacteria were maintained at −20 °C and −80 °C on 40% (v/v) glycerol.

The strains were periodically monitored by *16S rDNA* amplification followed by enzymatic restriction with *AluI* [67,68], and by *P. savastanoi* PCR specific assays [69].

### 3.2. Molecular Techniques and DNA Sequences Analysis

Standard general recombinant DNA techniques were performed according to Sambrook *et al.* [70]. Genomic DNA was purified from bacterial liquid cultures (OD_600_ = 0.8) using Gentra^®^ Puregene kit (Qiagen, Valencia, CA, USA), according to manufacturers' instructions. DNA amplicons were purified from agarose gels with PureLink^®^ Quick Gel Extraction Kit (Invitrogen Inc., Carlsbad, CA, USA), under the conditions recommended by the manufacturer. DNA concentration was evaluated both spectrophotometrically, with NanoDrop™ ND-1000 (NanoDrop Technologies Inc., DE, USA), and visually by standard agarose gel electrophoresis [1% agarose (w:v) in TBE 1X] [70].

Oligonucleotide primers for overlapping amplicons targeting all the *hrp/hrc* genes were designed according to the data available on the main databases on *P. savastanoi* and on bacteria taxonomically related, and then used to screen the genomic libraries of strains *Psv5*, *Psn23* and *Psf134* by PCR. Primers were designed by using Beacon Designer 8.0 software (Premier Biosoft International, Palo Alto, CA, USA) (Table S4). For each library, those clones giving positive amplification signals with all the primer pairs here used were identified. Among those, one clone for each *P. savastanoi* pathovar was selected and isolated (clones ST-5-22-4A, ST-23-19-2F and ST-134-31-7G for *Psv5*, *Psn23* and *Psf134*, respectively).

The nucleotide sequences of the entire TTSS clusters of *Psv5*, *Psn23* and *Psf134* were then determined by sub-cloning and double-strand sequencing the library clones (Eurofins MWG Operon Ltd., Ebersberg, Germany).

DNA sequences were annotated with the aid of Basic Local Alignment Search Tool (BLAST) [71], and predicted proteins were analyzed with the online Structure Prediction Suite SOSUI [31-34].

The presence of insertion sequences was assessed through ISfinder database [72,73].

Predicted proteins were analyzed for the presence of conserved domains and for homologies, by BLAST-CD search [35] and by ClustalW2 software [36], respectively.

### 3.3. Phylogenetic Analysis

A phylogenetic analysis was performed on the complete TTSS cluster of the bacterial strains listed in Table 1 [37-45] and on each of its major operons (*hrpRS*, *hrpZ*, *hrpC*, *hrpU* and *hrpJ*). The analysis involved seventeen nucleotide sequences that were aligned using ClustalW2 software [36], with the “Slow” Pairwise Alignment Type, and manually adjusted, if needed. The evolutionary history was inferred by using the Maximum Likelihood method based on the Tamura-Nei model, with the aid of Mega software ver. 5 [74-76]. The congruence of branching order was assessed with 1,000 bootstrap replications. Initial trees for the heuristic search were automatically obtained as follows. If the number of common sites was <100 or less than one fourth of the total number of sites, the maximum parsimony method was used. Otherwise, BIONJ method with MCL distance matrix was used. The tree is drawn to scale, with branch lengths related to the number of substitutions per site. All the positions containing gaps and missing data were eliminated.

Phylogenetic congruence between trees was inferred by SH test [50] with dnaML software contained in the Phylip package (ver 3.69) [77]. This test quantitatively evaluates the likelihood of alternative trees given a data set and basically establishes if the evolutionary histories between different data sets are the same.

### 3.4. Quantitative Real-Time PCR Analysis

Starter liquid cultures of strains *Psv*5, *Psn*23 and *Psf*134 were grown overnight in 20 mL KB, at 26 °C and under continuous shaking at 100 rpm. Cells were washed in sterile physiological solution (0.85% NaCl in distilled water) twice and used to inoculate 6 mL of fresh KB and MM medium, adjusting the concentration at 0.5 OD_600_. The cultures were then incubated as above, sampled after 3 h, 6 h, 18 h and 24 h, and used for RNA extraction performed with RNeasy Midi Kit (Qiagen). About 1 μg of RNA for each treatment was reverse transcribed using QuantiTect Reverse Transcription Kit (Qiagen), according to the manufacturer's instructions.

Quantitative Real-Time PCR (RT-qPCR) was carried out on iQ5 Cycler—Real-Time PCR Detection System (Bio-Rad Laboratories, Inc., Hercules, CA, USA), in 96 well PCR plates, with 25 μL reaction mixture volume, using iQ SYBR^®^ Green Supermix (Bio-Rad), according to the manufacturer's instructions. The primers used for Real-Time expression analysis are reported in Table 3. As housekeeping gene, *16S rDNA* was used. Each sample was run in triplicate, including standards and negative controls, using three different batches of cDNA obtained from three independent RNA preparations. The PCR protocol was 40 cycles with 95 °C for 20 seconds, 60 °C (or 62 °C exclusively for *hrpV* gene) for 20 seconds, and 72 °C for 20 seconds, after an initial step of 95 °C for 3 min. The amount of fluorescence given by incorporation of the SYBR Green dye into double-stranded DNA was evaluated for each sample at the end of each cycle, and analyzed to determine the resulting threshold cycle (Ct) values by iQ5 Optical System Software 2.0 (Bio-Rad). Dissociation analysis of amplicons was performed (from 60 to 95 °C, with a 0.5 °C increase every 5 seconds) at the end of each PCR run to check for aspecific amplifications. The comparative Livak (2^−ΔΔCT^) method [60] was used to analyze the mRNA level. The Ct values of each gene tested were normalized to the Ct values of the housekeeping gene, to obtain relative expression data for each gene examined.

In order to avoid significant measurement inaccuracies, the amplification efficiency of each primer pair was estimated producing Real-Time PCR curves for a ten-fold dilution series of *Psn23* genomic DNA (from 50 ng to 0.5 pg) used as template. The slope of the log-linear phase of each curve reflects the amplification efficiency, which should range between 90% and 100%, with slope value between −3.2 and −3.4, and R^2^ of at least 0.998.

All the data obtained represent the mean of three independent replication ± SD. Statistical analyses were performed using the two-tailed t-test.

## 4. Conclusions

For the first time here the TTSS clusters of the pathovars *nerii* and *fraxini* of the species *P. savastanoi* were sequenced. Moreover the TTSS sequence of another *Psv* strain was also obtained, in addition to that of *Psv* strain NCPPB3335 whose genome was recently published. The availability of these data provided an important opportunity to carry out a more robust phylogenetic and statistical analysis than has occurred in the past on the evolution of this secretion system during the differentiation of the species belonging to the *P. syringae* complex.

By comparing the congruence among the sequences of the entire TTSS and its five major operons from seventeen species and pathovars of this complex, here we found that operons *hrpZ* and *hrpRS* have been the most affected by the dynamical processes occurring in the shaping of this system during the adaptive evolution of these bacteria to their hosts. The positive selection acting on some structural genes belonging to *hrpZ* operon, such as *hrpA*, was reasonably functional to avoid recognition by the host defenses. For a phytopathogenic bacterium, it is also important to be able to finely regulate *hrp*/*hrc* gene expression to successfully interact with its hosts. The incongruence between *hrpRS* data, and any of the other trees but its own, supports the hypothesis that the evolution of this operon was crucial for the adaptive process of these bacteria to new hosts. Moreover, according to our results the evolution of TTSS across *P. syringae* bacteria was quite coherent with that of the core genome and of the rest of the flexible genome.

In this regard, differential patterns were found for the *in vitro* expression of four *hrp*/*hrc* genes under inducing conditions in the three *P. savastanoi* pathovars examined here. According to our hypothesis, this could be related to a pathovar-specific regulation of TTSS genes. Further studies are needed to ascertain if this occurs also *in planta*, and has any role in the determination of the host-range of these *P. savastanoi* pathovars.

## Supplementary Material

**Table S1:** Characteristics of Hrc predicted proteins of *P. savastanoi* TTSS.

**Name**		**length (aa)**	**ave. hyd.**		**TM-Reg**	**sign**	**loc**	**Ccoil reg.**	**CDDs**	**Homology (%)**
HrcC	*Psf*	699	−0,326609	(S)	-	+ (1–23)	OM	307–334	+	99.0
	*Psv*	699	−0,326609	(S)	-	+ (1–23)	OM	307–334	+	99.0
	*Psn*	699	−0,319456	(S)	-	+ (1–23)	OM	307–334	+	99.0
HrcJ	*Psf*	268	0.216418	(M)	2 (P1–23 / P220–242)	-	IM	26–58, 91–127	+	98.0
	*Psv*	268	0.211567	(M)	2 (P1–23 / P220–242)	-	IM	26–58, 91–127	+	99.0
	*Psn*	268	0.211567	(M)	2 (P1–23 / P220–242)	-	IM	26–58, 91–127	+	99.0
HrcN		449	0.003341	(S)	-	-	C	202–231, 358–406	+	99.0
HrcQa		238	−0.123950	(S)	-	-	C	121–157, 207–238	+	98.0
HrcQb		129	−0.571317	(S)	-	-	C	-	+	100.0
HrcR	*Psf*	208	0.719712	(M)	4 (P8–30 / P44–66 / P150–172 / P179–201)	-	IM	62–103	+	100.0
	*Psv*	208	0.719712	(M)	4 (P8–30 / P44–66 / P150–172 / P179–201)	-	IM	62–103	+	100.0
	*Psn*	208	0.710577	(M)	4 (P8–30 / P44–66 / P150–172 / P179–201)	-	IM	62–103	+	99.0
HrcS		88	1,061364	(M)	3 (P10–32 / S42–64 / S67–88)	-	IM	-	+	100.0
HrcT		264	0.936364	(M)	7 (S12–34 / S43–65 / P76–98 / S134–156 / P186–208 / P217–239 / P243–264)	+ (1–37)	IM	-	+	99.0
HrcU		359	0.140111	(M)	5 (S26–48 / P73–95 / S132–154 / S157–179 / P183–205)	-	IM	201–248	+	99.0
HrcV		695	0.266475	(M)	6 (P31–53 / S71–93 / P108–130 / P203–225 / S237–259 / P296–318)	+ (1–17)	IM	473–563	+	99.0

Columns, ordered from left to right indicate: (1) Putative protein name; (2) Name of the corresponding pathovar when non synonymous mutations are present; (3) Length of putative protein; (4) Average of hydrophobicity, according to SOSUI [31]; (5) S = Soluble, M = Membrane, as predicted by SOSUI [31]; (6) Presence, number and type (P = Primary, S = Secondary) of Trans-Membrane regions, predicted by SOSUI [31]; (7) Presence of Signal Peptide, predicted by SOSUIsign [32]; (8) Localization of putative protein (EC = Extracellular, C = Cytoplasm, OM = Outer Membrane, P = Periplasm, IM = Inner membrane, U = Unknown), predicted by SOSUIgramN [33]; (9) Presence of Coiled-coil regions, predicted by SOSUIcoil [34]; (10) Presence of Conserved Domains, evaluated using BLAST-CDDs [35]; (11) Percentage of homology with the same predicted protein from *P. syringae* pv. *phaseolicola* 1448A, evaluated with ClustalW [36].

**Table S2:** Characteristics of Hrp predicted proteins of *P. savastanoi* TTSS.

**Name**		**length (aa)**	**ave. hyd.**		**TM-Reg**	**sign**	**loc**	**Ccoil reg.**	**CDDs**	**Homology (%)**
HrpA		108	−0.331482	(S)	-	-	EC	59–105	+	97.0
HrpB		124	−0.184678	(S)	-	-	EC	52–90	+	99.0
HrpD		176	−0.253977	(S)	-	-	C	-	-	97.0
HrpE		193	−0.180311	(S)	-	-	C	34–99	+	100.0
HrpF		74	−0.393243	(S)	-	-	C	-	+	100.0
HrpG		146	0.042466	(S)	-	-	C	-	-	98.0
HrpJ		368	−0.305978	(S)	-	+ (1–30)	OM	30–76, 82–157, 197–228, 245–278, 330–368	+	99 0
HrpL	*Psf*	184	−0.438587	(S)	-	-	C	152–179	+	98.0
	*Psv*	184	−0.43858	(S)	-	-	C	152–180	+	98.0
	*Psn*	184	−0.460870	(S)	-	-	C	152–181	+	98.0
HrpO		148	−1.135810	(S)	-	-	C	10–148	+	99.0
HrpP		190	−0.461053	(S)	-	-	P	138–165	+	98.0
HrpQ		324	−0.169445	(S)	-	-	C	220–250	+	99.0
HrpR		306	−0.113072	(S)	-	-	C	261–304	+	99.0
HrpS	*Psf*	302	−0.106954	(S)	-	-	C	105–142, 246–286	+	99.0
	*Psv*	302	−0.098344	(S)	-	-	C	105–132, 246–286	+	99.0
	*Psn*	302	−0.106954	(S)	-	-	C	105–142, 246–286	+	99.0
HrpT		67	−0.167164	(M)	+ (P1–23)	+ (1–29)	U	-	-	100.0
HrpV	*Psf*	115	−0.217391	(S)	-	-	C	40–73	-	99.0
	*Psv*	115	−0.217391	(S)	-	-	C	40–73	-	100.0
	*Psn*	115	−0.205218	(S)	-	-	C	40–80	-	99.0
HrpZ		347	−0.275792	(S)	-	+ (1–23)	EC	24–62	+	98.0

Columns, ordered from left to right indicate: (1) Putative protein name; (2) Name of the corresponding pathovar when non synonymous mutations are present; (3) Length of putative protein; (4) Average of hydrophobicity, according to SOSUI [31]; (5) S = Soluble, M = Membrane, as predicted by SOSUI [31]; (6) Presence, number and type (P = Primary, S = Secondary) of Trans-Membrane regions, predicted by SOSUI [31]; (7) Presence of Signal Peptide, predicted by SOSUIsign [32]; (8) Localization of putative protein (EC = Extracellular, C = Cytoplasm, OM = Outer Membrane, P = Periplasm, IM = Inner membrane, U = Unknown), predicted by SOSUIgramN [33]; (9) Presence of Coiled-coil regions, predicted by SOSUIcoil [34]; (10) Presence of Conserved Domains, evaluated using BLAST-CDDs [35]; (11) Percentage of homology with the same predicted protein from *P. syringae* pv. *phaseolicola* 1448A, evaluated with ClustalW [36].

**Table S3:** Real-Time gene expression analysis for selected TTSS genes of *P. savastanoi*.

***Normalized expression (n-fold)* ± sd ***				

	***Psv* 5**			

	3 h	6 h	18 h	24 h

***hrpA***	10.2 ± 1.4	9.9 ± 0.8	31.1 ± 4.1	124.7 ± 9.9
***hrpS***	14.8 ± 0.9	22.4 ± 2.0	37.3 ± 3.3	152.1 ± 11.1
***hrpL***	11.8 ± 0.9	15.8 ± 2.3	29.9 ± 2.0	134.7 ± 8.7
***hrpV***	4.4 ± 0.2	53.2 ± 6.3	19.5 ± 1.5	32.1 ± 3.4

	***Psn* 23**			

	3 h	6 h	18 h	24 h

***hrpA***	18.33 ± 2.1	21.34 ± 3.36	54.59 ± 6.43	23.97 ± 3.2
***hrpS***	41.7 ± 3.9	24.43 ± 2.9	50.65 ± 7.23	14.6 ± 2.2
***hrpL***	29.87 ± 3.11	4.99 ± 0.98	11.2 ± 1.4	22.4 ± 2.1
***hrpV***	78.99 ± 8.45	21.3 ± 3.43	7.78 ± 0.9	7.66 ± 1.1

	***Psf* 134**			

	3 h	6 h	18 h	24 h

***hrpA***	134.78 ± 10.9	90.23 ± 7.98	4.89 ± 1.1	0.98 ± 0.23
***hrpS***	31.21 ± 4.22	27.33 ± 4.3	5.23 ± 1.43	1.21 ± 0.4
***hrpL***	6.11 ± 1.98	40.23 ± 2.34	3.5 ± 0.12	1.11 ± 0.03
***hrpV***	7.68 ± 2.31	7.98 ± 2.1	5.4 ± 0.45	78.92 ± 6.15

* Normalized fold increase in the expression of each analyzed gene in *Psv5*, *Psn23* and *Psf134* grown in MM *vs.* KB at 3, 6, 18 and 24 h after bacterial inoculation on fresh medium. Standard deviations (sd) are reported.

**Table S4:** Primers used for *P. savastanoi* TTSS cluster sequencing.

**Primer name**	**Primer sequence (5′–3′)**
Hrp For1	ATGAGCACAGACATTGAT
Hrp For2	TCACCCAGGAGATTGCCGC
Hrp For3	CAAATCTGGGGATCGTCG
Hrp For4	ATGTCTGCGTGATCGCTTCG
Hrp For5	ATCAAGGAGTTGCAGATCTG
Hrp For6	ATGGCGCTCGTTGTGATCC
Hrp For7	TCGGCACTCGACATCATCG
Hrp For8	TATCTTCACCGCCGCAAC
Hrp For9	TGAACTGCGGCCTGACACTC
Hrp For10	CAAAATAACCTCGACAGCAC
Hrp For11	TCAGGAATACCACTTCCAGT
Hrp For12	AACCCATCCATTCTTACCC
Hrp For13	AAGAGAGCGAGGTGTATATCG
Hrp For14	GCGAACCACTTTGTAGATGT
Hrp For15	CCAGGGTGATTTCACTGAC
Hrp For16	CATGCCCAGCATGAATTG
Hrp For17	AGACTGCTCTGCAGCTTGT
Hrp For18	ACCAGGACTCGTCCACAG
Hrp For19	GAACGGTTTTGTGCGAAG
Hrp For20	GGTGACATTGCTGAGGATCC
Hrp For21	GCGTACATTCCTGAGTGAAA
Hrp For22	GCACTGAAGGCCACTCAT
Hrp For23	GTGAGCACTCGCTGTATCTC
Hrp For24	ACACGCCGATGGAAATAC
Hrp For25	GGTCTGGTTCACGTTCATT
Hrp For26	TTTTCATAGGACGGTTCTGA
Hrp For27	CAACCATGCGTATATCCAGT
Hrp For28	CTCAAACAGTCGTCCAACAT
Hrp For29	AGGCGGGCTTCCTCAGCCAG
Hrp For30	CTTGCAAACCGACCTGGC
Hrp For31	TATCCACCATGCTCGCCAAC
Hrp Rev1	GAGTTGCAGATCTGATTT
Hrp Rev2	GCACCGCTTTCCGAGCAT
Hrp Rev3	GGATTGACCGGGCGCATTGA
Hrp Rev4	GATAGCCAGTCGTCACAGCGT
Hrp Rev5	CCTCGACAGCACGCTGAAT
Hrp Rev6	GATGTAGGCCTGCTGGATA
Hrp Rev7	AATATCGAGCCCATCACCGCCGGG
Hrp Rev8	GCTGTTCGCTACCCTGTCG
Hrp Rev9	CTTGTCAGCCAGATGCTCT
Hrp Rev10	CGTATCTGTTTGGGGGTAGC
Hrp Rev11	CATATTGATGAACTGAATCAGCTC
Hrp Rev12	ACCTGCTGAACGCCAATT
Hrp Rev13	ACCACGCCGTATCTGAAC
Hrp Rev14	CGGTGTGGCATGCACTAC
Hrp Rev15	GAGTCCTGCTCGATCAGC
Hrp Rev16	ATCTTGCATTCCAGCAGAAT
Hrp Rev17	CCTTCTTCAGCGTTCAGT
Hrp Rev18	CGAACAACTGACTTTCCTTG
Hrp Rev19	CAACAACGTCGTCACGTG
Hrp Rev20	TGTAGTGATAAAAACGGCGT
Hrp Rev21	GTGGTGATCAGGCCTTTGTGC
Hrp Rev22	CCAATATGAGCGAGTGGAT

## References

[1] Tampakaki A.P., Skandalis N., Gazi A.D., Bastaki M.N., Sarris P.F., Charova S.N., Kokkinidis M., Panopoulos N.J. (2010). Playing the “Harp”: Evolution of our understanding of *hrp*/*hrc* genes. Annu. Rev. Phytopathol..

[2] Jones J.D., Dangl J.L. (2006). The plant immune system. Nature.

[3] Grant S.R., Fisher E.J., Chang J.H., Mole B.M., Dangl J.L. (2006). Subterfuge and manipulation: Type III effector proteins of phytopathogenic bacteria. Annu. Rev. Microbiol..

[4] Block A., Li G., Fu Z.Q., Alfano J.R. (2008). Phytopathogen type III effector weaponry and their plant targets. Curr. Opin. Plant Biol..

[5] Zhou J.M., Chai J. (2008). Plant pathogenic bacterial type III effectors subdue host responses. Curr. Opin. Plant Biol..

[6] Guo M., Tian F., Wamboldt Y., Alfano J.R. (2009). The majority of the type III effector inventory of *Pseudomonas syringae* pv. *tomato* DC3000 can suppress plant immunity. Mol. Plant Microbe Interact..

[7] Huynh T.V., Dahlbeck D., Staskawicz B.J. (1989). Bacterial blight of soybean: Regulation of a pathogen gene determining host cultivar specificity. Science.

[8] Rahme L.G., Mindrinos M.N., Panopoulos N.J. (1992). Plant and environmental sensory signals control the expression of *hrp* genes in *Pseudomonas syringae* pv. *phaseolicola*. J. Bacteriol..

[9] Xiao Y., Lu Y., Heu S., Hutcheson S.W. (1992). Organization and environmental regulation of the *Pseudomonas syringae* pv. *syringae* 61 *hrp* cluster. J. Bacteriol..

[10] Xiao F., Goodwin S.M., Xiao Y., Sun Z., Baker D., Tang X., Jenks M.A., Zhou J.M. (2004). *Arabidopsis* CYP86A2 represses *Pseudomonas syringae* type III genes and is required for cuticle development. EMBO J..

[11] Ortiz-Martín I., Thwaites R., Macho A.P., Mansfield J.W., Beuzón C.R. (2010). Positive regulation of the Hrp type III secretion system in *Pseudomonas syringae* pv. *phaseolicola*. Mol. Plant Microbe Interact..

[12] Ortiz-Martín I., Thwaites R., Mansfield J.W., Beuzón C.R. (2010). Negative regulation of the Hrp type III secretion system in *Pseudomonas syringae* pv. *phaseolicola*. Mol. Plant Microbe Interact..

[13] Taira S., Tuimala J., Roine E., Nurmiaho-Lassila E.L., Savilahti H., Romantschuk M. (1999). Mutational analysis of the *Pseudomonas syringae* pv. *tomato hrpA* gene encoding Hrp pilus subunit. Mol. Microbiol..

[14] Bretz J., Losada L., Lisboa K., Hutcheson S.W. (2002). Lon protease functions as a negative regulator of type III protein secretion in *Pseudomonas syringae*. Mol. Microbiol..

[15] Thwaites R., Spanu P.D., Panopoulos N.J., Stevens C., Mansfield J.W. (2004). Transcriptional regulation of components of the type III secretion system and effectors in *Pseudomonas syringae* pv. *phaseolicola*. Mol. Plant Microbe Interact..

[16] Haapalainen M., van Gestel K., Pirhonen M., Taira S. (2009). Soluble plant cell signals induce the expression of the Type III Secretion System of *Pseudomonas syringae* and upregulate the production of pilus protein HrpA. Mol. Plant Microbe Interact..

[17] Tang X., Xiao Y., Zhou J.M. (2006). Regulation of the Type III Secretion System in phytopathogenic bacteria. Mol. Plant Microbe Interact..

[18] MacLean D., Studholme D.J. (2010). A Boolean model of the *Pseudomonas syringae hrp* regulon predicts a tightly regulated system. PLoS ONE.

[19] Preston G., Deng W.L., Huang H.C., Collmer A. (1998). Negative regulation of *hrp* genes in *Pseudomonas syringae* by HrpV. J. Bacteriol..

[20] Hendrickson E.L., Guevera P., Ausubel F.M. (2000). The alternative sigma factor RpoN is required for *hrp* activity in *Pseudomonas syringae* pv. *maculicola* and acts at the level of *hrpL* transcription. J. Bacteriol..

[21] Lan L., Deng X., Xiao Y., Zhou J.M., Tang X. (2007). Mutation of Lon protease differentially affects the expression of *Pseudomonas syringae* Type III Secretion System genes in rich and minimal media and reduces pathogenicity. Mol. Plant Microbe Interact..

[22] Mole B.M., Baltrus D.A., Dangl J.L., Grant S.R. (2007). Global virulence regulation networks in phytopathogenic bacteria. Trends Microbiol..

[23] Sisto A., Cipriani M.G., Morea M. (2004). Knot formation caused by *Pseudomonas syringae* subsp. *savastanoi* on Olive plants is *hrp*-dependent. Phytopathology.

[24] Pérez-Martínez I., Rodríguez-Moreno L., Lambertsen L., Matas I.M., Murillo J., Tegli S., Jiménez A.J., Ramos C. (2010). Fate of a *Pseudomonas savastanoi* pv. *savastanoi* Type III Secretion System mutant in Olive plants (*Olea europaea L*.). Appl. Environ. Microbiol..

[25] Han C.G., Shiga Y., Tobe T., Sasakawa C., Ohtsubo E. (2001). Structural and functional characterization of IS679 and IS66-family elements. J. Bacteriol..

[26] Xiao Y., Hutcheson S.W. (1994). A single promoter sequence recognized by a newly identified alternate sigma factor directs expression of pathogenicity and host range determinants in *Pseudomonas syringae*. J. Bacteriol..

[27] Petnicki-Ocwieja T., van Dijk K., Alfano J.R. (2005). The *hrpK* operon of *Pseudomonas syringae* pv. *tomato* DC3000 encodes two proteins secreted by the type III (Hrp) protein secretion system: HopB1 and HrpK, a putative type III translocator. J. Bacteriol..

[28] Alfano J.R., Collmer A. (1997). The type III (Hrp) secretion pathway of plant pathogenic bacteria: Trafficking harpins, Avr proteins, and death. J. Bacteriol..

[29] He S.Y., Nomuraa K., Whittam T.S. (2004). Type III protein secretion mechanism in mammalian and plant pathogens. Biochim. Biophys. Acta.

[30] Rodríguez-Palenzuela P., Matas I.M., Murillo J., López-Solanilla E., Bardaji L., Pérez-Martinez I., Rodríguez-Moskera M.E., Penyalver R., López M.M., Quesada J.M. (2010). Annotation and overview of the *Pseudomonas savastanoi* pv. *savastanoi* NCPPB 3335 draft genome reveals the virulence gene complement of a tumour-inducing pathogen of woody hosts. Environ. Microbiol..

[31] Hirokawa T., Boon-Chieng S., Mitaku S. (1998). SOSUI: Classification and secondary structure prediction system for membrane proteins. Bioinformatics.

[32] Gomi M., Sonoyama M., Mitaku S. (2004). High performance system for signal peptide prediction: SOSUIsignal. Chem-Bio Inform. J..

[33] Imai K., Asakawa N., Tsuji T., Akazawa F., Ino A., Sonoyama M., Mitaku S. (2008). SOSUI-GramN: High performance prediction for subcellular localization of proteins in Gram-negative bacteria. Bioinformation.

[34] Tanizawa H., Taniguchi M., Ghimire G.D., Mitaku S. (2009). Prediction of fragile points of coiled coils. Chem-Bio Inform J..

[35] Marchler-Bauer A., Lu S., Anderson J.B., Chitsaz F., Derbyshire M.K., DeWeese-Scott C., Fong J.H., Geer L.Y., Geer R.C., Gonzales N.R. (2011). CDD: A Conserved Domain Database for the functional annotation of proteins. Nucleic Acids Res..

[36] Thompson J.D., Higgins D.G., Gibson T.J. (1994). CLUSTALW: Improving the sensitivity of progressive multiple sequence alignment through sequence weighting, position-specific gap penalties and weight matrix choice. Nucleic Acids Res..

[37] Baltrus D.A., Nishimura M.T., Romanchuk A., Chang J.H., Mukhtar M.S., Cherkis K., Roach J., Grant S.R., Jones C.D., Dangl J.L. (2011). Dynamic evolution of pathogenicity revealed by sequencing and comparative genomics of 19 *Pseudomonas syringae* isolates. PLoS Pathog..

[38] Green S., Studholme D.J., Laue B.E., Dorati F., Lovell H., Arnold D., Cottrell J.E., Bridgett S., Blaxter M., Huitema E. (2010). Comparative genome analysis provides insights into the evolution and adaptation of *Pseudomonas syringae* pv. *aesculi* on *Aesculus hippocastanum*. PLoS ONE.

[39] Joardar V., Lindeberg M., Jackson R.W., Selengut J., Dodson R., Brinkac L.M., Daugherty S.C., Deboy R., Durkin A.S., Giglio M.G. (2005). Whole-genome sequence analysis of *Pseudomonas syringae* pv. *phaseolicola* 1448A reveals divergence among pathovars in genes involved in virulence and transposition. J. Bacteriol..

[40] Feil H., Feil W.S., Chain P., Larimer F., DiBartolo G., Copeland A., Lykidis A., Trong S., Nolan M., Goltsman E. (2005). Comparison of the complete genome sequences of *Pseudomonas syringae* pv. *syringae* B728a and pv. *tomato* DC3000. Proc. Natl. Acad. Sci. USA.

[41] Alfano J.R., Charkowski A.O., Deng W.L., Badel J.L., Petnicki-Ocwieja T., van Dijk K., Collmer A. (2000). The *Pseudomonas syringae* Hrp pathogenicity island has a tripartite mosaic structure composed of a cluster of type III secretion genes bounded by exchangeable effector and conserved effector loci that contribute to parasitic fitness and pathogenicity in plants. Proc. Natl. Acad. Sci. USA.

[42] Studholme D.J., Ibanez S.G., MacLean D., Dangl J.L., Chang J.H., Rathjen J.P. (2009). A draft genome sequence and functional screen reveals the repertoire of type III secreted proteins of *Pseudomonas syringae* pathovar *tabaci* 11528. BMC Genomics.

[43] Song E.S., Park Y.J., Chae S.C., Kim J.G., Cho H.J., Lee G.B., Lee B.M. (2006). Construction of a bacterial artificial chromosome library and characterization of *hrp/hrc* gene cluster of *Pseudomonas syringae* pathovar *tagetis* LMG5090. Biotechnol. Lett..

[44] Buell C.R., Joardar V., Lindeberg M., Selengut J., Paulsen I.T., Gwinn M.L., Dodson R.J., Deboy R.T., Durkin A.S., Kolonay J.F. (2003). The complete genome sequence of the *Arabidopsis* and tomato pathogen *Pseudomonas syringae* pv. *tomato* DC3000. Proc. Natl. Acad. Sci. USA.

[45] Araki H., Tian D., Goss E.M., Jakob K., Halldorsdottir S.S., Kreitman M., Bergelson J. (2006). Presence/absence polymorphism for alternative pathogenicity islands in *Pseudomonas viridiflava*, a pathogen of *Arabidopsis*. Proc. Natl. Acad. Sci. USA.

[46] Sawada H., Suzuki F., Matsuda I., Saitou N. (1999). Phylogenetic Analysis of *Pseudomonas syringae* pathovars suggests the horizontal gene transfer of *argK* and the evolutionary stability of *hrp* gene cluster. J. Mol. Evol..

[47] Guttman D.S., Gropp S.J., Morgan R.L., Wang P.W. (2006). Diversifying selection drives the evolution of the type III secretion system pilus of *Pseudomonas syringae*. Mol. Biol. Evol..

[48] Gardan L., Shafik H., Belouin S., Broch R., Grimont F., Grimont P.D. (1999). DNA relatedness among the pathovars of *Pseudomonas syringae* and description of *Pseudomonas tremae* sp. nov. and *Pseudomonas cannabina* sp. nov. (ex Sutic and Dowson 1959). Int. J. Syst. Bacteriol..

[49] Bull C.T., Manceau C., Lydon J., Kong H., Vinatzer B.A., Fischer-Le Saux M. (2010). *Pseudomonas cannabina* pv. *cannabina* pv. nov., and *Pseudomonas cannabina* pv. *alisalensis* (Cintas Koike and Bull, 2000) comb. nov., are members of the emended species *Pseudomonas cannabina* (ex Sutic & Dowson 1959) Gardan, Shafik, Belouin, Brosch, Grimont & Grimont 1999. Syst. Appl. Microbiol..

[50] Shimodaira H., Hasegawa M. (1999). Multiple comparisons of log-likelihoods with applications to phylogenetic inference. Mol. Biol. Evol..

[51] Gropp S., Guttman D. (2004). The PCR amplification and characterization of entire *Pseudomonas syringae hrp/hrc* clusters. Mol. Plant Pathol..

[52] Brown E.W., Allard M.W., van der Zwet T. (1998). Phylogenetic characterization of the eubacterial *lcrD* gene family: Molecular evolutionary aspects of pathogen-induced hypersensitivity in plants. Cladistics.

[53] Naum M., Brown E.W., Mason-Gamer R.J. (2008). Phylogenetic evidence for extensive horizontal gene transfer of type III secretion system genes among enterobacterial plant pathogens. Microbiology.

[54] Oh C.H., Beer S.V. (2005). Molecular genetics of *Erwinia amylovora* involved in the development of fire blight. FEMS Microbiol. Lett..

[55] Jovanovic M., James E.H., Burrows P.C., Rego F.G.M., Buck M., Schumacher J. (2011). Regulation of the co-evolved HrpR and HrpS AAA+ proteins required for *Pseudomonas syringae* pathogenicity. Nat. Commun..

[56] O'Brien H.E., Thakur S., Guttman D.S. (2011). Evolution of plant pathogenesis in *Pseudomonas syringae*: A genomics perspective. Annu. Rev. Phytopathol..

[57] Sarkar S.F., Guttman D.S. (2004). Evolution of the core genome of *Pseudomonas syringae*, a highly clonal, endemic plant pathogen. Appl. Environ. Microbiol..

[58] King E.O., Ward M.K., Raney D.E. (1954). Two simple media for the demonstration of pyocyanin and fluorescein. J. Lab. Clin. Med..

[59] Livak K.J., Schmittgen T.D. (2001). Analysis of relative gene expression data using real-time quantitative PCR and the 2^−ΔΔCT^ method. Methods.

[60] Büttner D., Bonas U. (2006). Who comes first? How plant pathogenic bacteria orchestrate type III secretion. Curr. Opin. Microbiol..

[61] Chatterjee A., Cui Y., Yang H., Collmer A., Alfano J.R., Chatterjee A.K. (2003). GacA, the response regulator of a two-component system, acts as a master regulator in *Pseudomonas syringae* pv. *tomato* DC3000 by controlling regulatory RNA, transcriptional activators, and alternate sigma factors. Mol. Plant Microbe Interact..

[62] Grimm C., Aufsatz W., Panopoulos N.J. (1995). The *hrpRS* locus of *Pseudomonas syringae* pv. *phaseolicola* constitutes a complex regulatory unit. Mol. Microbiol..

[63] Schuster M., Grimm C. (2000). Molecular Domain switching between *hrpR* and *hrpS* affects the regulatory function of the hybrid genes in *Pseudomonas syringae* pv. *phaseolicola*. Plant Pathol..

[64] Hutcheson S.W., Bretz J., Sussan T., Jin S., Pak K. (2001). Enhancer-binding proteins HrpR and HrpS interact to regulate *hrp*-encoded type III protein secretion in *Pseudomonas syringae* strains. J. Bacteriol..

[65] Daniell S.J., Delahay R.M., Shaw R.K., Hartland E.L., Pallen M.J., Booy F., Ebel F., Knutton S., Frankel G. (2001). Coiled-coil domain of enteropathogenic *Escherichia coli* type III secreted protein EspD is involved in EspA filament-mediated cell attachment and hemolysis. Infect. Immun..

[66] Delahay R.M., Frankel G. (2002). Coiled-coil proteins associated with type III secretion systems: A versatile domain revisited. Mol. Microbiol..

[67] Caccamo D., di Cello F., Fani R., Gugliandolo C., Maugeri T.L. (1999). Polyphasic approach to the characterisation of marine luminous bacteria. Res. Microbiol..

[68] Sisto A., Cipriani M.G., Tegli S., Cerboneschi M., Stea G., Santilli E. (2007). Genetic characterization by fluorescent AFLP of *Pseudomonas savastanoi* pv. *savastanoi* strains isolated from different host species. Plant Pathol..

[69] Tegli S., Cerboneschi M., Marsili Libelli I., Santilli E. (2010). Development of a versatile tool for the simultaneous differential detection of *Pseudomonas savastanoi* pathovars by End Point and Real-Time PCR. BMC Microbiol..

[70] Sambrook J., Fritsch E.F., Maniatis T.A. (1989). Molecular Cloning: A Laboratory Manual.

[71] Altschul S.F., Gish W., Miller W., Myers E.W., Lipman D.J. (1990). Basic local alignment search tool. J. Mol. Biol..

[72] Altschul S.F., Madden T.L., Schäffer A.A., Zhang J., Zhang Z., Miller W., Lipman D.J. (1997). Gapped BLAST and PSI-BLAST: A new generation of protein database search programs. Nucleic Acids Res..

[73] Siguier P., Perochon J., Lestrade L., Mahillon J., Chandler M. (2006). ISfinder: The reference centre for bacterial insertion sequences. Nucleic Acids Res..

[74] Tamura K., Peterson D., Peterson N., Stecher G., Nei M., Kumar S. (2011). MEGA5: Molecular evolutionary genetics analysis using maximum likelihood, evolutionary distance, and maximum parsimony methods. Mol. Biol. Evol..

[75] Tamura K., Nei M. (1993). Estimation of the number of nucleotide substitutions in the control region of mitochondrial DNA in humans and chimpanzees. Mol. Biol. Evol..

[76] Sneath P.H.A., Sokal R.R. (1963). Numerical Taxonomy.

[77] Felsenstein J. (1989). PHYLIP-phylogeny inference package (Version 3.2). Cladistics.

